# Methoprene-Induced Genes in Workers of Formosan Subterranean Termites (*Coptotermes formosanus* Shiraki)

**DOI:** 10.3390/insects11020071

**Published:** 2020-01-21

**Authors:** He Du, Reina L. Tong, Xueyi Huang, Bingrong Liu, Runmei Huang, Zhiqiang Li

**Affiliations:** 1 Guangdong Key Laboratory of Animal Conservation and Resource Utilization, Guangdong Public Laboratory of Wild Animal Conservation and Utilization, Guangdong Institute of Applied Biological Resources, Guangzhou 510260, China; duh@giabr.gd.cn (H.D.); liubr@giabr.gd.cn (B.L.); HRMtermites@163.com (R.H.); 2 Department of Entomology and Nematology, Ft. Lauderdale Research and Education Center, Institute of Food and Agricultural Sciences, University of Florida, 3205 College Ave., Ft. Lauderdale, FL 33314, USA; reinat@ufl.edu

**Keywords:** caste regulation, juvenile hormone, methoprene bioassay

## Abstract

Termites have a distinct polyphenism controlled by concise hormonal and molecular mechanisms. Workers undergo double molts to transform into soldiers (worker–presoldier–soldier). Juvenile hormone analogs, such as methoprene, can induce workers to transform into presoldiers. However, the molecular mechanism underlying the worker-to-presoldier transformation in *Coptotermes formosanus* Shiraki is still not clear. We sequenced the transcriptome of workers four days after they had fed on methoprene-treated filter paper and control group workers, which fed on acetone-treated filter paper. The transcriptome of *C. formosanus* was assembled using the *de novo* assembly method. Expression levels of unigenes in the methoprene-treated group and the control group were compared. The differentially expressed genes were further analyzed by Gene Ontology (GO) term enrichment analysis and Kyoto Encyclopedia of Genes and Genomes pathway enrichment analysis. Tetrapyrrole binding, oxidoreductase activity, and metal ion binding were the only three enriched GO terms. Juvenile hormone synthesis was the first ranked enriched pathway. Carbohydrate, amino acid, and lipid metabolism pathways were also enriched. These three pathways may be related to fat body development, which is critical for presoldier formation. Our results have demonstrated the significance of JH synthesis pathways, and pathways related to fat body development in the artificial induction of presoldiers.

## 1. Introduction

All termites are eusocial. Termites have distinct polyphenism, in which the members of the colony have different morphologies to accomplish the division of labor. In contrast with the holometabolous social insects, in which the haplodiploid system controls the sex development within the colony, termites are hemimetabolous, and all colony members are diploid and have the same genetic background. The caste regulation mechanism in Hymenopteran insects, such as honey bees, has been widely studied from hormone to pheromone, and from genetic control to epigenetic studies [1,2,3]. However, our knowledge on the genetic and epigenetic control of caste differentiation in termites is relatively limited. Termites have a very complex polyphenic system. In lower termites (evolutionarily basal), the developmental pathway is linear, meaning that pseudergates/workers can develop into alates (winged reproductives) and soldiers; however, in higher termites (termites in the family Termitidae), the developmental pathway to alates and soldiers is bifurcated at the early stages of development [4]. Pseudergates refer to immature lower termites that function as workers, but may later differentiate into soldiers or reproductives. How the same genomic information is expressed differently in the later stages of development is still unclear and needs to be further studied.

The soldier caste is sterile and has adaptations for the defense of the colony. In lower termites, pseudergates/workers develop into soldiers, while in higher termites, it is the larvae that develop into soldiers [4]. Juvenile hormone (JH) plays a central role in the differentiation of the soldier caste in termites. Application of juvenile hormone III (JH III) or juvenile hormone analog (JHA) can induce workers to differentiate into presoldiers, which molt again into soldiers. After application of JHA, the hemolymph JH III decreases initially, but gradually increases afterwards [5]. The most obvious research method to reveal the molecular mechanism underlying soldier caste differentiation is to compare the gene expression profiles during the developmental stages. Initially, scientists used the differential display and microarray method and found a series of key genes differentially expressed, such as the genes encoding SOL1, cuticle proteins, nucleic acid binding proteins, and hexamerins [6,7,8]. Currently, with the development of next-generation sequencing technology, RNA-Seq has been used to reveal the key genes in soldier formation. *TGF-β* and *Neural Lazarillo* were identified as the key genes controlling soldier formation [9,10]. The hormonal changes and the genetic cascade underlying the transition drives the transformation to presoldier. 

*Coptotermes formosanus* Shiraki is a worldwide pest [11]. JHAs were proposed as an active ingredient of termite baits, because the superfluous induction of presoldiers by JHAs could lead to inadequate nutrition supply, and even collapse of the colony [12,13,14]. Induction of presoldiers from workers by JHAs has been used to study the soldier caste differentiation mechanism in lower termites, such as *Hodotermopsis sjostedti* (Holmgren), *Zootermopsis nevadensis* (Hagen), and *Reticulitermes flavipes* (Kollar) [6,7,8,10,15,16,17,18,19,20,21]. Methoprene is considered one of the most effective JHAs to induce transformation from worker to presoldier in *C. formosanus* [22,23,24,25]. However, the presoldier ratio induced by methoprene is not high enough to lead the collapse of the colony, which limits the usage of JHAs as an effective termite bait in *C. formosanus*. Although JHAs have been widely tested as a candidate termite control agent, the molecular mechanism underlying the artificial transformation from worker to presoldier has seldom been studied in *C. formosanus*, with respect to the economic importance of this species. *C. formosanus* is a transition species between the lower termites and higher termites [26,27]. The JH signaling pathway has experienced changes during the evolution from higher termites to lower termites [28]. It is probable that *C. formosanus* has a special soldier caste differentiation mechanism compared to that of other lower termites. After treating workers with methoprene, presoldiers start to appear in about two weeks in *C. formosanus*. According to a histological study in *H. sjostedti*, fat body development is initiated the third day after treatment with JHA [29]. It is inferred that pseudergates/workers are bound to transform into presoldiers in the early days after JHA induction. In order to acquire a more advanced understanding of the soldier formation mechanism in *C. formosanus*, we compared the transcriptome between the workers in the early days after methoprene treatment and the control group. The objective of this study was to detect candidate genes that may be involved in soldier caste regulation.

## 2. Materials and Methods 

### 2.1. Methoprene Bioassay

Termites (*C. formosanus*) were collected in Shangchong Fruit Tree Park, Guangzhou, China, using the bucket trap method [30]. The workers (undifferentiated larvae of at least the third instar) were subjected to the methoprene bioassay as follows. Filter paper (32 mm in diameter) was treated with an acetone solution of methoprene (91% in purity, Raw Material Medicine Reagent Co. Ltd., Nanjing, China) to make the concentration 1000 ppm (this group is referred to as “M” herein). The control was made by adding only acetone to the filter paper (this group is referred to as “C” herein). Paired filter paper was put into the Petri dish (35 mm in diameter), and wetted with 240 μL of deionized water. Twenty workers were placed in one Petri dish, which was considered as one replicate. The Petri dishes were placed in a box, and a piece of wet paper towel was placed inside the box. The boxes were put in complete darkness at 28 °C. Termites used in the bioassay were collected from one colony. According to our preliminary experiment, the ratio of presoldiers can reach up to 42% ± 7% ($\bar{X}\pm \mathrm{SE}$, *n* = 5) after treatment with methoprene. Each treatment had three replicates. 

### 2.2. RNA Extraction and Sequencing

Four days after the methoprene bioassay, the workers were collected. The heads of the workers from one Petri dish were collected with a scalpel, so that influence of gut protozoa and bacteria were eliminated. These heads were then put into one Eppendorf tube and immersed in liquid nitrogen. Then, the specimens were stored at −80 °C. The RNA was extracted using the TRIzol^®^ regeant (Thermo Fisher Scientific, Waltham, MA, USA). The concentration and integrity of the RNA were checked using the Agilent 2100 RNA 6000 Nano kit. Then, cDNA libraries were constructed for each of the six samples using VAHTS^TM^ mRNA-Seq V3 Library Prep Kit for Illumina^®^ (Vazyme Biotech Co., Ltd., Nanjing, China). Sequencing was performed with Illumina HiSeq 2500.

### 2.3. De Novo Transcriptome Assembly and Annotation

The raw reads were filtered by removing the adapters, reads containing more than 10% unknown nucleotides, and low-quantity reads containing more than 50% low-quality (Q-value ≤ 20) bases. The ribosome RNA sequences of *C. formosanus* and the closely related species were retrieved from National Center for Biotechnology Information (NCBI), and integrated into the ribosome RNA database. The closely related species included *C. acinaciformis*, *C. gestroi*, *C. testaceus*, *C. niger*, and *C. sjostedti*. The filtered reads were then mapped to the ribosome RNA database using Bowtie2 [31]. The mapped reads were removed. The remaining reads of all six samples were combined and assembled using the Trinity package to get a reference transcriptome [32]. 

Annotation of the unigenes was made by searching the following database using BLASTX in Blast2GO using the reciprocal best hit approach (e value < 1e−5): NCBI nonredundant protein database (nr), SwissProt, Kyoto Encyclopedia of Genes and Genomes (KEGG), EuKaryotic Orthologous Groups (KOG), and Clusters of Orthologous Groups (COG) [33]. If a unigene matched multiple protein sequences, the sequence with the lowest e value was chosen as the optimal annotation; if the target sequences had the same e value, the sequence with the highest similarity score was considered as optimal annotation. 

### 2.4. Analysis of Samples Relationship

The expression level of the unigene was normalized by the RPKM (reads per kb per million reads) method [34]. To test stability and reliability of the operation, the RPKM of unigenes from one sample was correlated with that from the six samples using a Pearson correlation. Principal component analysis (PCA) was performed using R package gmodels [35]. PCA is used to decrease thousands of observations (the expression level of unigenes) into two distinct principal components to reveal the patterns of the samples. 

### 2.5. Analysis of Differentially Expressed Genes (DEGs) between Treatment

The EdgeR package was used to analyze the DEGs between M and C [36]. The fold change is defined as the ratio of the average expression level of a gene in the M group to that in the C group. If the fold change of a gene was more than 2 (|log_2_ (fold change)| > 1) and the false discovery rate (FDR) < 0.05, the gene was considered as differentially expressed. 

### 2.6. Validation of RNA-Seq Results

Eleven unigenes were randomly selected from the DEGs. A new bioassay with the same procedure as that of Section 2.1 was conducted to acquire the specimen for RNA extraction. The total RNA from the heads of the M group and the C group were extracted using TRIzol^®^ reagent (Thermo Fisher Scientific, Waltham, MA, USA). The total RNA was then transcribed into cDNA using the PrimeScript^®^ reagent Kit with gDNA Eraser (Perfect Real Time) (Takara, Dalian, China). The primers used for qPCR are shown in Table S1. The genes were quantified with the qPCR methods with PowerUp SYBR Green Master Mix (Thermo Fisher Scientific, Waltham, MA, USA). The qPCR was performed in CFX Connect Optics Module (Bio-Rad, Hercules, CA, USA). *RPS18* was used as the reference gene [37]. The fold change was calculated for each of the eleven unigenes, and then log_2_ transformed. The transformed fold change from the qPCR was correlated with that from the RNA-Seq using R [38].

### 2.7. GO and KEGG Pathway Enrichment Analysis

Gene Ontology (GO) is a standardized representation of gene and gene products for all species and has a controlled vocabulary to describe the attribute of the gene and the gene products. GO is composed of three parts: component, function, and process. The GO annotation was also performed for all the unigenes in order to conduct the GO enrichment analysis. KEGG is a database integrating the building blocks and the molecular interaction networks. The GO enrichment analysis and the KEGG pathway enrichment analysis were performed for all the DEGs to detect the enriched GO term and KEGG pathways. Fisher’s exact test was used for the analyses. The FDR adjusted *P* value (*Q* value) was used to determine significance (*Q* < 0.05).

## 3. Results

### 3.1. Sequencing Quality and De Novo Assembly of the Transcriptome

The sequencing quality is shown in Table 1. The clean reads totaled 121,622,855 bases. After the *de novo* assembly, 125,270 unigenes were acquired, whereas there were 30,955 in the NCBI nonredundant database (nr), 12,674 in the Swiss–Prot database, 11,901 in the EuKaryotic Orthologous Groups (KOG), and 12,121 in the KEGG pathway database. There were 31,391 unigenes with annotation. However, the rest of the 93,879 genes were still not annotated. The N50 of the unigenes was 2455 bp. The maximum length of the unigenes was 34,800 bp, the minimum 201 bp, and the average 970 bp.

### 3.2. Correlation of Samples

**Figure 1:**
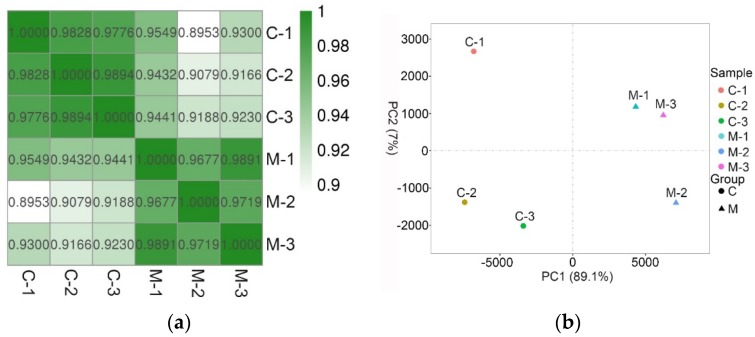
The correlation of samples. (**a**) Heatmap that shows the correlation coefficient among samples. The correlation of samples from the same groups was stronger than that from different groups; (**b**) Principal component analysis (PCA) of the six samples. PCA analysis showed that the samples in M group were more likely to cluster together, with a similar pattern in C group. See note for Table 1.

The heatmap of the correlation coefficient is shown in Figure 1a. According to the heatmap, samples in the C group were more likely to correlate with each other than with samples from the M group, and vice versa. According to the principal component analysis, the first principal component accounted for 89.1% of the variation, while the second component only accounted for 7% of the variation (Figure 1b).

### 3.3. DEGs between Treatments

According to the analysis of differences between groups, 2547 unigenes were differentially expressed (Table S2). Compared with the gene expression levels in C group, there were 1480 upregulated genes and 1067 downregulated genes in the M group.

### 3.4. Validation of RNA-Seq Results

**Figure 2:**
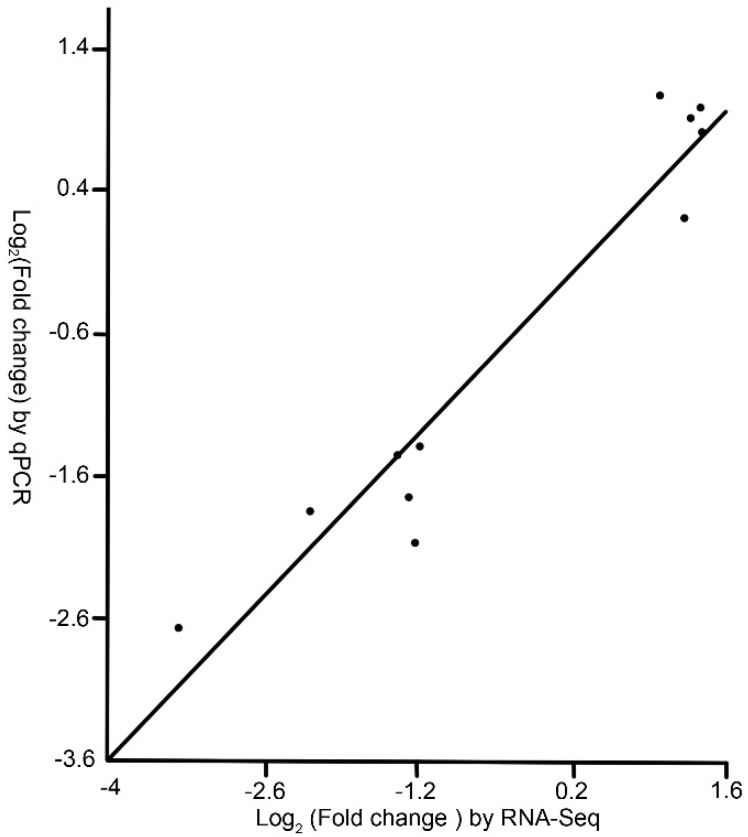
Correlation between qPCR data and RNA-Seq data. Each point represents the ratio of the average expression level of a gene in the M group to that in the C group. The data were log_2_ transformed. See note for Table 1.

The fold changes of genes calculated by the qPCR data were correlated to that of the RNA-Seq data. There was a strong correlation between RNA-Seq data and qPCR data (*r* = 0.9017, *P* < 0.0001), which demonstrated the reliability of the RNA-Seq results (Figure 2).

### 3.5. Gene Ontology (GO) Enrichment Analysis

**Figure 3:**
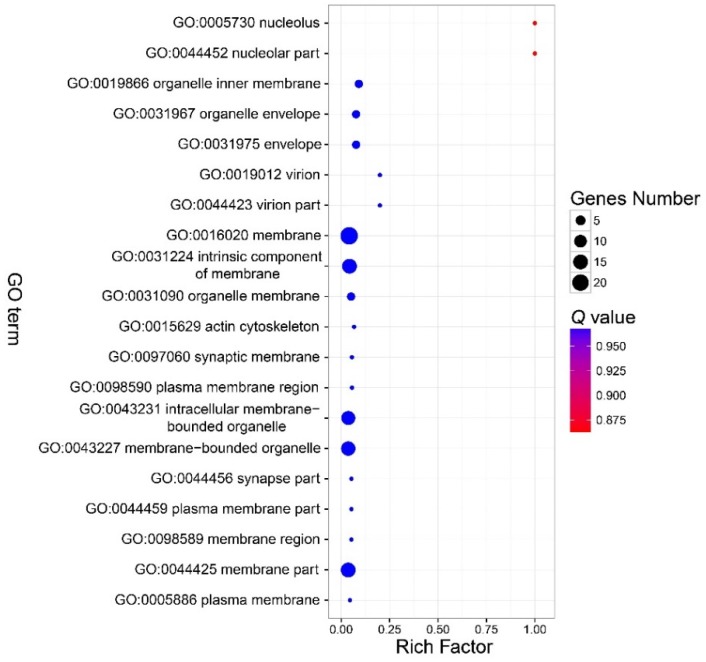
Bubble chart that shows the top 20 enriched GO terms in the cellular component part. No GO terms were enriched in this part. Note: The y-axis is the name of the GO term, the x-axis is the rich factor. Rich factor is the ratio of the number of the DEGs in a GO term to the number of total genes annotated to the GO term.

**Figure 4:**
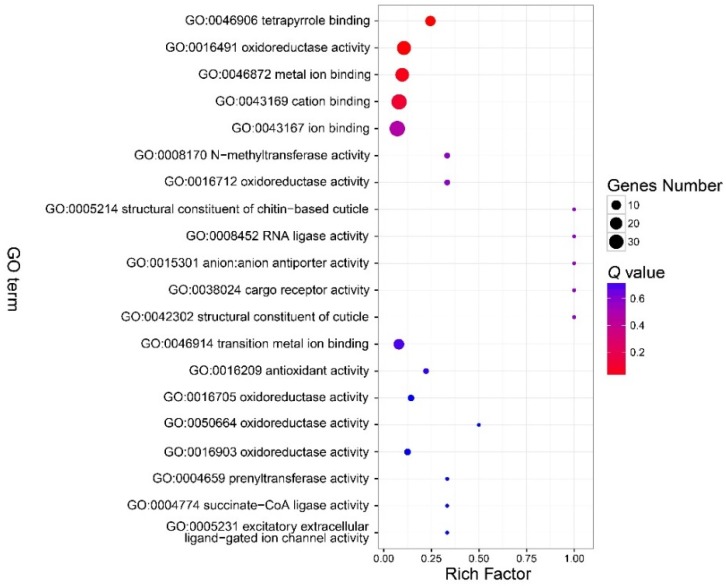
Bubble chart that shows the top 20 enriched GO terms in the molecular function part. There were three GO terms in the function part enriched. See note for Figure 3.

**Figure 5:**
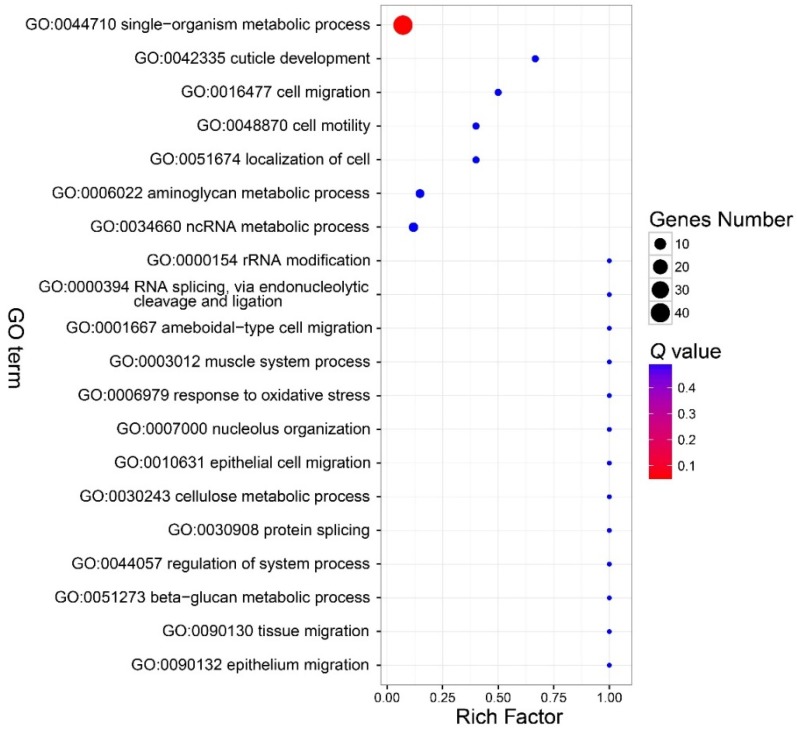
Bubble chart that shows the top 20 enriched GO terms in the biological process part. There were also no parts enriched in this part. See note for Figure 3.

There were no GO terms significantly enriched in the component part (Figure 3, Table S3). In the function part, three GO terms were enriched, and included GO:0046906 (tetrapyrrole binding) (*Q* < 0.01), GO:0016491 (oxidoreductase activity) (*Q* = 0.0006), and GO:0046872 (metal ion binding) (*Q* = 0.036) (Figure 4, Table S4). The DEGs in the enriched GO terms of the function part included *cytochrome P450*, *cytochrome c oxidase* (*COX*), etc. (Table S5). A number of GO terms (25) were significantly enriched, according to the *P* value. However, after adjustment of the *P* value, there were also no GO terms enriched in the process part (Figure 5, Table S6). 

### 3.6. KEGG Pathway Enrichment Analysis

**Figure 6:**
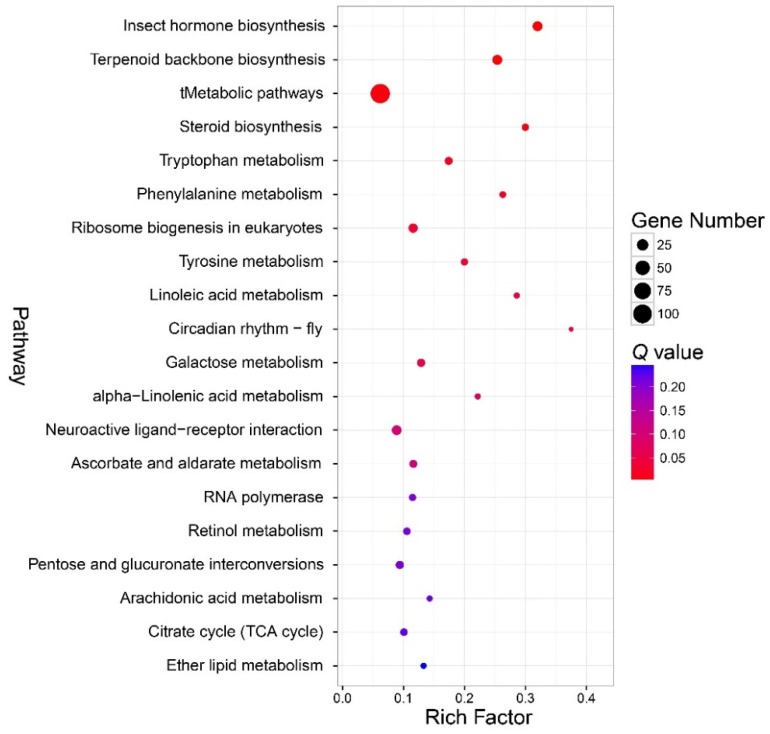
Bubble chart that shows the top 20 enriched pathways according to the Kyoto Encyclopedia of Genes and Genomes (KEGG) enrichment analysis. Note: the y-axis is the name of the pathway, the x-axis is the rich factor. Rich factor is the ratio of the number of the differentially expressed genes (DEGs) in a pathway to the number of total genes annotated to the pathway.

According to the KEGG pathway enrichment analysis, the enriched pathway was categorized into amino acid metabolism, carbohydrate metabolism, environmental adaptation, global and overview maps, lipid metabolism, metabolism of terpenoids and polyketides, and translation (Figure 6, Table S7). The most enriched pathway was the insect hormone biosynthesis pathway (*Q* < 0.01). Other pathways enriched mainly belonged to the biosynthesis of terpenoid and metabolic pathway (tMetabolic pathways (*Q* = 0.006)), and metabolism of amino acids (tryptophan metabolism (*Q* = 0.02), phenylalanine metabolism (*Q* = 0.03), tyrosine metabolism (*Q* = 0.003)), terpenoid backbone biosynthesis, (*Q* < 0.01), steroid biosynthesis (*Q* = 0.006), ribosome biogenesis in eukaryotes (*Q* = 0.003), linoleic acid metabolism (*Q* = 0.004), circadian rhythm-fly (*Q* = 0.046), and galactose metabolism (*Q* = 0.046). The DEGs in the enriched KEGG pathways are shown in Table S8.

## 4. Discussion

The transformation from pseudergate/worker into soldier requires a presoldier stage. The transformation from worker to presoldier involves a striking morphological change, with the most prominent change being the elongation of the mandibles. The mandibles of the presoldier look like that of the soldier, which is used for the defense of the colony. Using the RNA-Seq method, through comparing the group treated with methoprene for four days and the control group, we identified the early response genes after workers fed on methoprene in *C. formosanus*. Because there is no genome available for *C. formosanus*, the *de novo* assembly method was used to acquire a reference transcriptome by assembling all the reads from the six samples. The credibility of the RNA-Seq data was confirmed by the qPCR data. The stability and reliability of the operation can be shown through the small difference among the three samples in the same treatment. Through KEGG pathway enrichment analysis and the GO enrichment analysis, the enriched KEGG pathway and GO component were identified. 

JH has a significant role in insect growth, development, diapause, and reproduction. As is widely documented in literature, JH also plays a key role in the formation of soldier castes [5,39,40,41]. After topical application of JHA on pseudergates of *H. sjostedti*, the JH titer decreased at first, but gradually increased and reached the plateau just before the pseudergate-presoldier molt [5]. According to our RNA-Seq results, the JH synthesis pathway is the most significantly enriched pathway, which was consistent with the RNA-Seq study in *Z. nevadensis* [42]. The changes of expression profiles of the JH synthesis genes were also documented in *Z. nevadensis* [43]. The alteration of the expression of JH synthesis genes reflected the change of the titer of JH in the hemolymph, and the change drove the transformation of workers into presoldiers. Based on our data, another significantly enriched pathway was the terpenoid backbone biosynthesis. Both the JH synthesis and terpenoid backbone biosynthesis belong to the metabolism of terpenoids and polyketides. There are interactions between the JH synthesis pathway and the terpenoid backbone biosynthesis pathway. The change in the JH synthesis pathway conveys feedback signals to the terpenoid backbone biosynthesis. Terpenes may also be directly involved in soldier caste regulation. Two terpenes (γ-cadinene and γ-cadinenal) are reported acting as pheromone to regulate soldier caste differentiation [44]. In addition, the fact that JH III could not induce formation of presoldiers in *C. formosanus* also leads to speculation that other terpenoids besides JH III are involved in the worker-presoldier transformation. 

The JH receptor, methoprene-tolerant protein (Met), has basic helix-loop-helix (bHLH) and Per-Arnt-Sim (PAS) domains. It heterodimerizes with other bHLH–PAS factors and transducts the signal by upregulating the expression of *Krüppel homolog 1* [45,46]. Because of JH’s significant role in soldier formation, it is inferred that the JH signaling pathway is involved in the formation of soldiers. Met was suggested to play a regulation role in soldier morphogenesis [17]. However, since the JH signal pathway is not in the KEGG pathway, it does not show up in the “results part” of the KEGG enrichment analyses. Circadian rhythm-fly was significantly enriched according to the KEGG pathway enrichment analysis. Period is an important member of the circadian rhythm-fly pathway, and it contains the PAS domain [47]. It is probable that Period interacts with Met through the PAS domain to regulate soldier formation. Other pathways, such as the FoxO signaling pathway, which have been reported to have cross-talk with the JH signaling pathway [48], may also be involved in the regulation of presoldier formation. However, the FoxO signaling pathway was not enriched (Table S7), although *FOXO* was differentially expressed between the M group and the C group (Table S2). 20-hydroxyecdysone (20E) signaling pathway is required for the accomplishment of molting. Expression of ecdysone synthesis genes increases three days after the appearance of the first instar larvae in *Z. nevadensis* [42]. It takes around eight days for the first appearing third instar larvae to molt into a presoldier [49], but at least ten days for the artificial induction of presoldiers in *C. formosanus* [25]. Our current study only focused on the early stage of the worker-presoldier molt, the activation of the FoxO signaling pathway and 20E signaling pathway may be found in the later stage of the worker-presoldier molt in *C. formosanus*. This is probably the reason the FoxO signaling pathway and 20E pathway were not enriched in the KEGG pathway enrichment analysis. The TGFβ signaling pathway acts like a mediation between the JH and 20E signaling pathways in *Z. nevadensis* [10]. The finding above was acquired in termites under natural conditions. Whether *TGFβ* plays a similar function in the artificial induction of soldiers in *C. formosanus* should be confirmed.

The most prominent histological change during worker-to-presoldier transformation is the development of fat body; the abdominal cavity is filled with fat body four days before molting [29]. Granules, which are constituted of proteins, also appeared, accompanying fat body development in *H. sjostedti* [29]. The fat body functions as the nutrient storage site. Proteins, glycogen, and lipids are the three major nutritional components inside the fat body. The changes at the histological level were consistent with our RNA-Seq data. Several KEGG pathways related to carbohydrate, amino acid, and lipid metabolism, were enriched according to our RNA-Seq data. Besides the construction materials, the sites in which the protein was synthesized was also under construction, which was implied by the enrichment of the ribosome-related gene pathways. The upregulation of those metabolic pathways reflected the histological change after the application of JHA. The enrichment of metabolic pathways was also reported in *Z. nevadensis* [42]. The fat body plays an important role in insect metamorphosis. The development of the fat body may lay the foundation for worker-to-presoldier metamorphosis. The energy and construction materials stored in the fat body before molting is mobilized for metamorphosis. Insulin/insulin-like growth factor signaling (IIS) is essential to insect metamorphosis and fat body development [50]. Given the important role of the fat body in presoldier morphogenesis, it is inferred that IIS plays a key role in the process. The components of the insulin signaling pathway - insulin receptor protein kinase B - was demonstrated to regulate presoldier morphogenesis during mandible elongation [16]. However, the insulin signal pathway was not enriched in the KEGG enrichment analysis either. Since the sample was taken at the early stage of the transformation process, it is plausible that the IIS play a part in the later stage of the process.

In the GO enrichment analysis, there were only three GO terms enriched, all of which were in the function part. They were tetrapyrrole binding, oxidoreductase activity, and metal ion binding. After examining genes in those parts, we found that *COX* and *cytochrome P450* were major genes. Variation in the activity of COX accompanies the insect development and caste differentiation. Different expression levels of *COXIII* were observed among larvae, workers, nymphs, and soldiers of *Reticulitermes santonensis* (Kollar) [51]. The expression level of *COX* is different between the delates and alates of *Solenopsis invicta* Buren [52]. P450 is a heme-containing monooxygenase that can oxidize steroids, fatty acids, and xenobiotics. P450 is key to insect development. It has interactions with both the JH synthesis and 20E metabolism pathways. The last enzyme in the JH synthesis pathway methyl farnesoate epoxidase is CYP15A1 [53]. P450 genes are critical in the JH-dependent transition from worker to presoldier. Expression of *CYP6AM1* is downregulated during the artificial induction of worker to presoldier [15]. Another P450 gene, *CYP15F1*, is upregulated during the artificial induction of worker to presoldier [18]. *CYP15F1* is involved in presoldier formation [18]. According to our RNA-Seq results, some P450 genes were upregulated, while others downregulated. The function of those P450 genes deserves future research. Since the signaling pathway related to fatty acids was enriched, and P450 is also involved in fatty acid biosynthesis, whether P450 serves an important function in fatty acid metabolism needs to be confirmed. Since P450 is also involved in xenobiotics oxidation, it is also speculated that P450 may be involved in the oxidation of methoprene.

The molecular study of *C. formosanus* still lags behind and does not match its economic importance. The genome of *C. formosanus* has not been sequenced yet. Therefore, the annotation of the transcriptome may not be accurate. Because of the economic importance of this termite species, more genomic studies need to be conducted for this species. The transcriptome at the early stage of soldier development was sequenced. The early responsive genes were identified. The identification of the response gene by RNA-Seq data laid the foundation for future research of soldier differentiation mechanisms in *C. formosanus*. One thing that needs to be mentioned is that only one colony was used. There is variability among different colonies in *C. formosanus*. Thus, the findings in our research need to be confirmed in other colonies. Although only one point sample after methoprene treatment was chosen to sequence the transcriptome, our data already reveal a convincing piece of information that energy was stored and construction materials were prepared. The hormonal change drives the transformation from worker to presoldier. The signal transduction pathways give the instructions to the cells inside the body and modulate the transformation process. The enriched GO terms and KEGG pathways shed light on the molecular mechanism of soldier differentiation. However, the function of those genes has not yet been confirmed by other molecular technologies, such as RNAi and qPCR. Those genes belong to the early response genes; future research will be conducted to identify the late response genes that drive the transformation from workers to presoldiers.

## 5. Conclusions

Through comparing the transcriptome of workers feeding on methoprene-treated filter paper and workers feeding on acetone-treated filter paper, methoprene-induced genes in workers were identified. The DEGs included genes in the JH synthesis pathway and JH signaling-related pathway (such as genes in the circadian rhythm-fly pathway). COX, P450 and genes in the carbohydrate, amino acid, and lipid metabolism pathways were also enriched. The DEGs play an important role in the dynamic change of JH titer, transduction of JH signaling, and fat body development. Our results demonstrated the importance of JH and fat body development in the soldier caste differentiation.

## Figures and Tables

**Table 1:** Summary of the sequencing quality of six samples.

Sample	Reads Len	GC	Adapter (%)	Low Quality (%)	Before Filter				After Filter			
					Reads Num	Data (bp)	Q20 (%)	Q30 (%)	Reads Num (%)	Data (bp)	Q20 (%)	Q30 (%)
C-1	150	42.42%	65,366 (0.15%)	370,632 (0.43%)	43,436,706	6,515,505,900	6,358,215,678 (97.59%)	6,082,493,543 (93.35%)	43,186,024 (99.42%)	6,406,109,175	6,269,904,593 (97.87%)	6,003,693,525 (93.72%)
C-2	150	42.74%	77,716 (0.20%)	332,628 (0.42%)	3,9552,300	5,932,845,000	5,805,725,329 (97.86%)	5,579,018,441 (94.04%)	39,308,270 (99.38%)	5,821,866,687	5,713,502,183 (98.14%)	5,496,049,001 (94.40%)
C-3	150	42.56%	73,332 (0.15%)	395,232 (0.41%)	48,329,198	7,249,379,700	7,101,010,753 (97.95%)	6,830,421,183 (94.22%)	48,058,250 (99.44%)	7,132,398,497	7,004,858,972 (98.21%)	6,743,894,191 (94.55%)
M-1	150	42.37%	59,430 (0.14%)	325,132 (0.37%)	43,451,442	6,517,716,300	6,395,677,154 (98.13%)	6,165,076,910 (94.59%)	43,229,446 (99.49%)	6,420,441,241	6,315,469,623 (98.37%)	6,092,847,541 (94.90%)
M-2	150	42.46%	127,050 (0.28%)	500,052 (0.55%)	45,789,528	6,868,429,200	6,711,965,107 (97.72%)	6,442,092,243 (93.79%)	45,412,452 (99.18%)	6,734,642,039	6,602,981,101 (98.05%)	6,344,371,629 (94.21%)
M-3	150	42.78%	51,240 (0.12%)	307,992 (0.37%)	41,601,322	6,240,198,300	6,106,260,337 (97.85%)	5,861,841,825 (93.94%)	41,396,086 (99.51%)	6,147,435,726	6,030,263,909 (98.09%)	5,793,613,090 (94.24%)

Note: C = control group. M = methoprene-treated group. Workers in the control group were fed with acetone-treated filter paper; workers in the treated group were fed with methoprene-treated filter paper.

## Data Availability

The sequence data were submitted to GenBank Sequence Read Archive databases under the BioProject PRJNA577439.

## Supplementary Materials

The following are available online at https://www.mdpi.com/2075-4450/11/2/71/s1, Table S1: Primers used for validation of RNA-Seq results, Table S2: DEGs between methoprene-treated group and control group, Table S3: GO enrichment analysis for all the DEGs in the cellular component part, Table S4: GO enrichment analysis for all the DEGs in the molecular function part, Table S5: DEGs in the significantly enriched GO terms in the molecular function part, Table S6: GO enrichment analysis for all the DEGs in the biological process part, Table S7: KEGG pathway enrichment analysis for all the DEGs, Table S8: DEGs in the significantly enriched KEGG pathways.
